# Ravulizumab exposure in early pregnancy

**DOI:** 10.1007/s00277-025-06586-6

**Published:** 2025-10-13

**Authors:** Wolfgang Füreder, Sonja Granser, Andreas Repa, Alex Farr

**Affiliations:** 1 Department of Medicine I, Division of Hematology & Hemostaseology, Medical University of Vienna, Währinger Gürtel 18-20, Vienna, A-1090 Austria; 2 Department of Obstetrics and Gynecology, Division of Obstetrics and Feto-Maternal Medicine and Comprehensive Center for Pediatrics (CCP), Medical University of Vienna, Vienna, Austria; 3 Department of Pediatrics and Adolescent Medicine, Medical University of Vienna, Vienna, Austria

**Keywords:** Paroxysmal nocturnal hemoglobinuria, Pregnancy, Ravulizumab, Eculizumab

## Abstract

Before complement inhibitors were available, pregnancy in women with paroxysmal nocturnal hemoglobinuria (PNH) was associated with considerable mortality, mainly due to thromboembolism. The complement-C5 inhibitor eculizumab has decreased this risk and improved pregnancy outcomes in women with PNH. Eculizumab has now largely been replaced by the longer acting ravulizumab in non-pregnant PNH patients. We report on ravulizumab exposure in early pregnancy in a 33 years-old PNH patient. She had become pregnant while on ravulizumab therapy and was switched to eculizumab in gestational week 11. Thus, exposure to ravulizumab occurred in a sensitive period of embryogenesis. Pregnancy was uneventful with a normal organ scan, followed by caesarean section. The male infant had an Apgar score of 9/9/10, without signs of perinatal morbidity. Birth weight was 3580 g, body length 53 cm, and head circumference 35.5 cm. The infant was fully breastfed and appeared well when being discharged home on the fourth day of life. Together, the use of ravulizumab during pregnancy in our patient was not harmful for the child. More data are needed to determine whether ravulizumab is indeed a safe treatment option for PNH in pregnancy.

## Introduction

PNH is rare hematologic disorder caused by a deficiency of glycosyl-phosphatidyl-inositol linked complement regulating proteins on various blood cells [1]. In addition to anemia, the disease can also cause massive thrombophilia [1]. Historically, pregnancy in women with PNH was associated with a high mortality rate of up to 20%, mainly due to thromboembolism [2].

The complement C5-inhibitor eculizumab has led to a dramatic improvement in of outcome [3] and is now considered standard of care for pregnant women with PNH who require therapy [4]. In non-pregnant PNH patients, eculizumab has largely been replaced by the longer acting C5-inhibitor ravulizumab. The drug has a mean half-life of approximately 50 days corresponding to a dosing interval of 8 weeks, compared to 2 weeks for eculizumab [4–6]. Ravulizumab achieves its long half-life via recycling in endosomes that is mediated through increased binding of the drug to the neonatal Fc-receptor [6]. Whether ravulizumab poses a risk for fetus is unclear and publications on ravulizumab in pregnancy are so far limited to one abstract [7]. An observational study collecting data on ravulizumab pregnancies is registered at ClinicalTrials.gov [8], but is currently only being conducted in the United States. In addition, the trial has only started in December 2024 and will unlikely deliver results anytime soon. Therefore, data to help decide women and treating physicians on optimal PNH therapy in pregnancy are urgently needed.

We report on a 33 years-old PNH patient who became pregnant while on complement inhibition with ravulizumab.

## Case report

Our patient had suffered a deep venous thrombosis in 2015 and received anticoagulation with low-molecular-weight-heparin (LMWH). She was diagnosed with PNH in 2016 and eculizumab therapy was started in 2017. Anticoagulation was discontinued after initiation of specific PNH-therapy. In 2019 the patient was switched to ravulizumab (Table 1). She had considered pregnancy and was therefore advised that a switch back to eculizumab and an eight-months wash-out period for ravulizumab before a planned pregnancy are recommended [9].

**Table 1:** Range of PNH-clone size, hemoglobin, reticulocyte counts and LDH at diagnosis, during eculizumab- and ravulizumab therapy, pregnancy, delivery and postpartum

Event	Diagnosis		Pregnancy 02/2024 – 11/2024		Delivery and postpartum	
Drug	08/2016	Eculizumab 03/2017 – 08/2019	Ravulizumab 09/2019 – 05/2024	Eculizumab 05/2024 – 11/2024	Eculizumab 11/2024	Ravulizumab* 12/2024 – 02/2025
PNH-clone size %						
Granulocytes	83	88–94	95–98	98	nd	97
Monocytes	80	nd	93–97	97–98	nd	97
Erythrocytes	4	3–87	4–97	98	nd	97
Hb, g/dL	13,4	13.0–14.1	13.2–14.7	10.4–13.3	8.1–8.8	13.2
Reticulocytes, G/L	59.0	72.3–99.0	69.2–119.5	129.6–152.3	123.7–218.8	149.4
LDH, U/L	509	212–305	205–279	181–280	313–809	242

Patient was diagnosed with PNH in 2016 following a deep venous thrombosis. Remarkably, no anemia was present at diagnosis and most of the follow up despite a considerable PNH-clone size. During pregnancy hemoglobin levels decreased as expected in pregnancy, while LDH remained below 1.5 x upper limit normal (ULN, normal range <250 U/L) at all times. Postpartum the patient suffered from mastitis resulting in breakthrough hemolysis, that was controlled with antibiotic therapy. * Therapy was changed back from eculizumab to ravulizumab in 12/2024. Therefore, laboratory values under the influence of ravulizumab are presented from an examination following this switch in 02/2025.

**Fig. 1:**
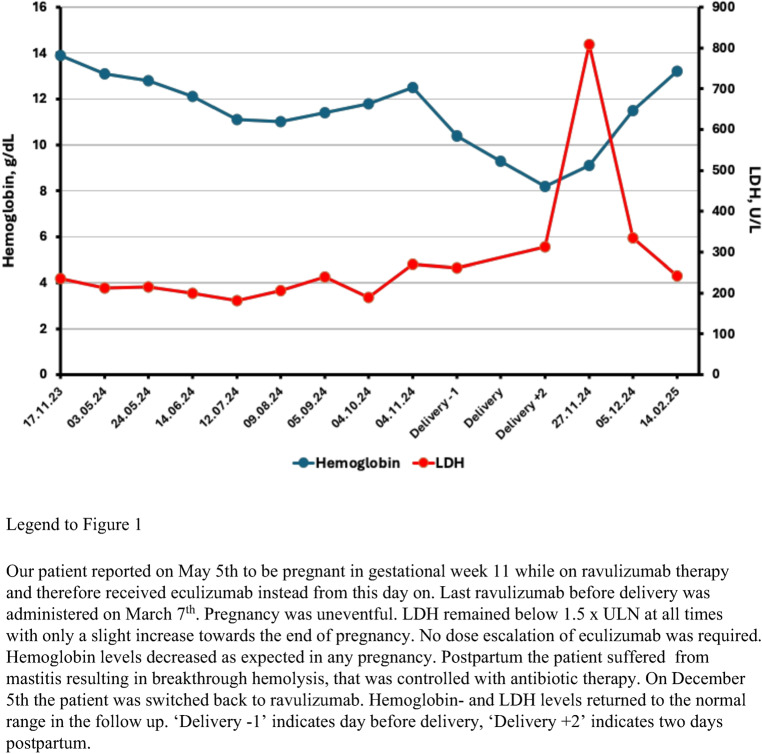
Hemoglobin and LDH levels before and during pregnancy and after delivery

In May 2024, the patient reported an unplanned pregnancy at gestational week 11. Thus, she had received the last dose of 3300 mg ravulizumab at gestational week 3 without knowing that she had been pregnant. The next dose of ravulizumab was therefore withheld and she was given eculizumab instead, starting with the induction protocol of 600 mg weekly for four weeks, then in week 5 and thereafter every two weeks 900 mg. Lactate-dehydrogenase (LDH) levels and blood counts remained stable after the switch from ravulizumab to eculizumab. While some patients may require increased dosage of eculizumab towards the end of pregnancy [4], in our patient standard dose was sufficient to keep LDH below 1.5 x upper limit of normal (ULN) (Fig. 1; Table 1).

Anticoagulation with LMWH in prophylactic dose of 4000 IU was started [4]. Due to retroplacental hematoma and vaginal bleeding at week 13, the LMWH dose was reduced to 2000 IU. In week 29 of gestational age, LMWH dose was increased to 4000 IU again. No further bleeding complications and no breakthrough hemolysis events occurred during pregnancy.

The patient was counselled during her pregnancy by hematologists and gynecologists. From an obstetric perspective, the patient had a normal first trimester and organ scan. Sonograms suggested a large for gestational age (LGA) fetus and led to an induction of labor at 39 weeks of pregnancy. Following the insertion of a vaginal prostaglandin insert, regular contractions led to increased vaginal bleeding and pathological fetal heart rate tracing. Therefore, a non-planned caesarean section was performed. The male infant showed physiological respiratory and cardiovascular adaptation (Apgar 9/9/10, umbilical artery pH 7.37), without any signs of perinatal morbidity. The infant’s anthropometry was symmetric and within the normal range (birth weight was 3580 g (0.33 standard deviation score (SDS)) at a body length of 53 cm (0.58 SDS) and a head circumference of 35.5 cm (0.18 SDS). The boy was fully breastfed and appeared well during the routine check-ups. Body temperature was between 36,5 and 37,5° Celsius. The postnatal weight loss was − 8% on the third day of life, and the infant gained weight (+ 70 g) on the fourth day of life when being discharged home.

Three weeks post-partum, we switched the mother back from eculizumab to ravulizumab therapy. LMWH prophylaxis was continued until 6 weeks post-partum. She provided written informed consent for the publication of this case report.

No ethics approval was required for this retrospective case report.

## Discussion

Published data regarding ravulizumab in pregnancy are so far limited to a series of six pregnancies in 5 patients [7]. Nonclinical reproductive toxicology studies have not been conducted with ravulizumab.

With eculizumab having proven to be safe and effective in pregnancy [3] ravulizumab has so far been largely avoided in these circumstances. IgG may cross the placental barrier and enter the fetal circulation [10]. Data with eculizumab indicate that eculizumab can indeed be found in cord blood samples [3]. It is not known whether this applies to ravulizumab as well. In our patient, the last dose of ravulizumab was administered in the third gestational week and therefore no traces of the drug would have been expected to be still present in the maternal (or fetal) circulation at the time of delivery.

Our patient received ravulizumab at early gestation without being aware to be pregnant at this time. Therefore, full exposure to ravulizumab occurred during a sensitive time-period of embryogenesis. Should ravulizumab be indeed teratogenic, one would expect to notice adverse effects in the child following exposure at this time of pregnancy. In our case, organ scans showed no abnormalities, and the infant adapted well without any ante-, peri- or postpartum morbidity. The LGA that had sonographically been suspected, was not confirmed postpartum.

Indeed, we have not observed any adverse effect of the fetal ravulizumab exposure in our case. Likewise, no abnormalities were detected in any of the 6 children exposed to ravulizumab reported by Höchsmann et al. [7]. One cannot rule out however, that fetal ravulizumab exposure might cause problems that may only become apparent later in life.

There are certain limitations to our observation. First, we report only a single case of ravulizumab exposure in pregnancy. Second, our patient and her child were exposed to ravulizumab only at the beginning of her pregnancy. Even when considering the long half-life of the drug, we are unable to provide any data on fetal ravulizumab exposure in later stages of pregnancy.

Together, we have not found any adverse effects on the infant that had been exposed to ravulizumab during early pregnancy. Data from a larger number of patients and their children are needed to establish whether fetal exposure to ravulizumab is indeed safe and unproblematic for the unborn.

## Data Availability

No datasets were generated or analysed during the current study.
